# Seroprevalence and risk factors for infection with *Bartonella bacilliformis* in Loja province, Ecuador

**DOI:** 10.1038/s41426-018-0110-5

**Published:** 2018-06-25

**Authors:** Shari L. Lydy, Mauricio S. Lascano, Josselyn E. Garcia-Perez, Amanda J. Williams-Newkirk, Mario J. Grijalva

**Affiliations:** 1 Centers for Disease Control and Prevention, Division of Vector-Borne Diseases, Rickettsial Zoonoses Branch, Atlanta, GA USA; 2 Department of Biomedical Sciences, Ohio University, Heritage College of Osteopathic Medicine, Infectious and Tropical Disease Institute, Athens, OH USA; 3 Pontificia Universidad Católica del Ecuador, Centro de Investigación para la Salud en América Latina, Escuela de Ciencias Biológicas, Quito, Ecuador; 4 Present Address: Organization for Tropical Studies-North American Branch, Tropical Diseases, Environmental Change and Human Health Program, Durham, NC USA; 5 Present Address: School of Medicine, Department of Microbiology and Immunology, University of Colorado, Denver, CO USA; 6 Present Address: Centers for Disease Control and Prevention, Division of Foodborne, Waterborne, and Environmental Diseases, Enteric Disease Laboratory Branch, Atlanta, GA USA

## Abstract

The seroprevalence and epidemiology of *Bartonella bacilliformis* infection in the Andean highlands of Ecuador is largely unknown. We conducted a sero-epidemiologic survey of 319 healthy children aged 1–15 years living in six rural, mountain communities in Loja Province, Ecuador. Blood was collected by finger stick onto filter paper and dried, and the eluted sera analyzed for antibodies to *B. bacilliformis* by rPap31 ELISA. Demographic, entomologic, and household variables were assessed to investigate associated risk factors for antibody seropositivity to *B. bacilliformis*. Seroprevalence of 28% was found among children in the study communities. Increased risk of seropositivity was associated with the presence of lumber piles near houses. Decreased risk of seropositivity was observed with the presence of animal waste and incremental 100 meter increases in elevation. Although investigation of clinical cases of Carrion’s disease was not within the scope of this study, our serology data suggest that infection of children with *B. bacilliformis* is prevalent in this region of Ecuador and is largely unrecognized and undiagnosed. This study highlights the need to further investigate the prevalence, pathogenesis, epidemiology, and disease impact of this pathogen in Ecuador.

## Introduction

Carrion’s disease in South America is caused by the flagellated, Gram-negative bacterium, *Bartonella bacilliformis* and humans are the only known reservoir of disease^1,2^. Initial infection (Oroya fever) typically results in extensive invasion of erythrocytes and causes fever, bacteremia, and severe hemolytic anemia. This is typically followed by a chronic cutaneous phase (verruga peruana) with secondary invasion of endothelial cells lining blood vessels and capillaries and the appearance of vascularized skin lesions^1,3,4^. Other forms of disease include mild febrile illness, verruga peruana in the absence of Oroya fever, or asymptomatic infection^5,6^.

Historically, Carrion’s disease is endemic in the inter-Andean mountain valleys of Peru, Ecuador, and Colombia at elevations of 600–3200 meters above sea level (MASL), presumably corresponding to the habitat of the sand fly vector *Lutzomyia verrucarum*^7–10^. However, infections with *B. bacilliformis* occur at lower elevations in the coastal lowlands and Amazonas in these countries, suggesting that other species of *Lutzomyia* may also be involved in transmission^11–14^. Previously, L. *maranonensis* and *L. robusta* were reported as potential vectors for Carrion’s disease in the region of Oriental del Maranon in Peru^12^. During an outbreak of Carrion’s disease in the Urubamba Valley of Peru in 1997, 1% of trapped *L. peruensis* were found to be naturally infected with *B. bacilliformis*^13^. Recently, *B. bacilliformis* was confirmed in *L maranonensis* in Cajamarca province in northern Peru by PCR^15^. *L. maranonensis* is present in Ecuador primarily in the Andean region (studies excluded Loja province)^16,17^ and would appear a likely vector for transmitting *B. bacilliformis* since both *L. verrucaram* and *L. peruensis* are absent from Ecuador^2,18^. Other *Lutzomyia* species found in Ecuador that may also be potential vectors of Carrion’s disease include *L. robusta*, *L. serrana*, and *L. ayacuchensis*^19–21^. *L. ayacuchensis* is found in the coastal lowlands of Manabi province and at 650–2500 MASL in the Andean highland provinces of Pinchincha, Chimborazo, and Azuay^16,22^. *L. robusta* is an Andean species found in Zumba province^4,12^, whereas its variant or closely related spp *L. serrana* is reported in coastal Manabi province^23,24^. Although several species of *Lutzomyia* are found in regions of Ecuador where *B. bacilliformis* infection has been reported, more studies are needed to unequivocally identify the vector(s) of Carrion’s disease there.

While Carrion’s disease is well documented in Peru^25,26^, there have been few published reports in Ecuador^2^. Sporadic cases of Oroya fever were previously reported in the mountain valleys of Zamora-Chinchipe Province bordering Peru, and verruga peruana was documented in the coastal lowland provinces of Manabí and Guayas on the Pacific coast of Ecuador^4,27,28^. We reported a case study of atypical infection with *B. bacilliformis* characterized by mild anemia and chronic splenomegaly in an expatriate Ecuadorian who visited northern Ecuador (provinces of Esmeraldas, Pichincha, and Sucumbios) where there were no previous reports of Carrion’s disease^29^. In 2009, several cases of verruga peruana were reported to us by a physician in Portoviejo, Manabi Province (G. Gutierrez, personal communication) thus confirming historical accounts of monophasic disease in the coastal lowlands of Ecuador.

Loja Province is a mountainous region bordered on the south by Piura Province in Peru and west by Zamora-Chinchipe Province in Ecuador where Oroya fever has occurred in the past. The aim of the current study is to determine the seroprevalence and risk factors of *B. bacilliformis* infection in children in remote, mountain communities located in central and southern Loja province.

## Results

**Fig. 1:**
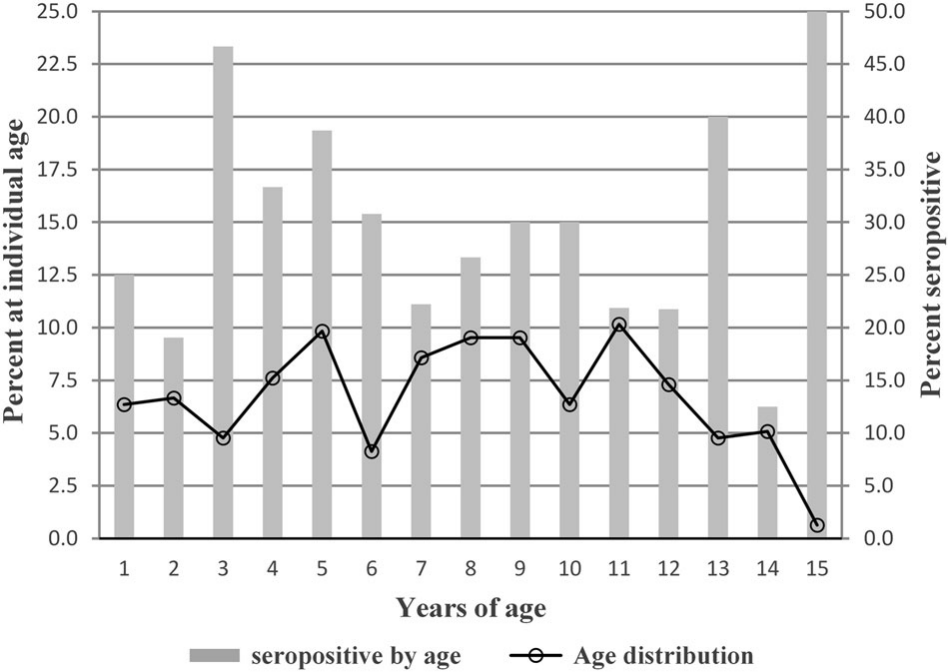
**Age curve of seropositivity showing the percentage of children at each age from total children in all six study communities in Loja Province compared to the percentage of seropositive children at each age.** The left axis corresponds to the line graph denoting the percentage of children at each age. The right axis corresponds to the bar graph denoting the percentage of children at each age that are seropositive

A total of 319 samples were collected from children in 128 households within six communities in Loja Province (Table 1). The six communities included approximately equal numbers of male and female children from 1–15 years of age and similar numbers of households per community (Tables 1 and 2). There were no statistical differences in age group distributions between communities (Fig. 1; *χ*^2^ = 6.2185, *p* = 0.7966). Likewise the percentages of children at each age (1–15) were similar and ranged from 5–10% of the study population except for age 15 that included only two individuals (Fig. 1). The average age of children in the study was 9 years old. There was also no association between seropositivity and gender (*M*^2^ = 0.0095; *p* *=* 0.9400; data not shown).

**Table 1:** Overall demographics and individual responses to entomological questions of surveyed children in the six communities in Loja province, Ecuador (EC)

	*n*	%
Gender		
Male	166	52.2
Female	153	47.8
Age categories, years		
1–5	112	35.1
6–10	119	37.3
11–15	88	27.6
Travel^a^		
None or within Loja province	256	82.6
Non-endemic provinces in EC	17	5.5
Endemic provinces in EC or Peru	26	8.4
Destination not stated	11	3.5
Recognize sand flies^b^		
Yes	168	57.1
No	126	42.9
Bitten by sand flies		
Yes	261	85.9
No	43	14.1

Community	# Houses	House elevations average MASL^c^	# Children per age group		
			1–5	6–10	11–15
Jacapo	27	1667	25	35	28
Usaime	26	1574	17	20	17
Galapagos	22	1268	22	20	18
Santa Rosa	14	1476	11	9	6
Vega del Carmen	20	1156	22	18	9
Chirimoyos	19	1112	14	18	10

^a^In Ecuador, non-endemic provinces included El Oro, Azuay, Sucumbios, and Pichincha (no reported Carrion’s disease). Endemic provinces in EC included Manabi and Guayas (verruga peruana) and Piura, Peru (Oroya fever)

^b^Sand flies were identified from pictures

^c^Meters above sea level (MASL)

**Table 2:** Overall *B. bacilliformis* seropositivity in children by community and age group

	Antibody positive by age group					Antibody negative by age group				
Community	1–5	6–10	11–15	Total	(%)^a^	1–5	6–10	11–15	Total	(%)^a^
Vega del Carmen	14^b^	7	4	25	51%	8	11	5	24	49%
Chirimoyos	6	9	5	20	48%	8	9	5	22	52%
Santa Rosa	5	4	2	11	42%	6	5	4	15	58%
Usaime	6	6	8	20	36%	12	14	9	35	64%
Galapagos	3	3	1	7	12%	19	17	17	53	88%
Jacapo	3	4	1	7	8%	22	31	27	80	92%
Total # of children per age group in all communities	37	32	21	90		75	87	67	229	
Total % Ab+ or Ab- children in all communities^c^	33%	27%	24%	28%		67%	73%	76%	82%	

Antibody was measured as IgG by rPap31 ELISA

^a^Percentage of antibody-positive or antibody-negative children per community

^b^Number of children in each age category

^c^Percentage of total antibody-positive or negative children by age group

The serologic results showed an average seroprevalence of 28% among children in the communities, ranging from 8% in Jacapo to 51% in Vega del Carmen (Table 2). Children at all ages (1–15) had antibodies to *B. bacilliformis*, with the highest rates of seropositivity at 3, 5, and 13 years of age. IgG antibody titers ranged from 64–1024, with a median titer of 128 and geometric mean titer (GMT) of 152.4 (data not shown).

The relationships between seropositivity and age groups (1–5, 6–10, 11–15) within and between communities were evaluated. Association of seropositivity and age groups within each community could not be tested because the numbers of children per each age group were too small (Table 2). When numbers of seropositive children within each age category were totaled and compared across all communities, there also was no association Table 2; (*M*^2^ = 0.9242; *p* *=* 0.6299).

However, there was a significant overall difference (*χ*^2^ = 48.33, *p* < 0.001) when comparing the total numbers of seropositive children between each community (Table 2). To determine which communities were contributing to this difference, post hoc pairwise testing was done using Fisher’s exact tests of independence. Briefly, all 15 possible pairwise comparisons between communities with seropositive children were done. For example, Chirimoyos (CY) was compared to Jacapo (JP), Galapagos (NGL), Vega del Carmen (NVC), Usaime (NVC), and Santa Rosa (SS) and so forth (Table 3). With 15 pairwise comparisons at a 95% confidence interval, the probability *p* *<* 0.003, obtained by dividing the 0.05 significance level by the number of pairwise comparisons (15), is significant. To summarize this data, the numbers of seropositive children were statistically different between communities for CY and JP, CY and NGL, JP and NVC, JP and NVS, JP and SS, and NGL and NVC (Table 3).

**Table 3:** Comparison of seropositive children between communities by post hoc pairwise testing using Fisher’s exact tests of independence

Community (Ab+)	JP (7)	NGL (7)	NVC (25)	NVS (20)	SS (11)
CY (20)^a^	< 0.0001^b^	0.0009	1.0	1.0	1.0
JP (7)		1.0	0.0001	0.0018	0.002
NGL (7)			0.0001	0.0299	0.0219
NVC (25)				0.843	1.0
NVS (20)					1.0

*CY* Chirimoyes, *JP* Jacapo, *NGL* Galapagos, *NVC* Vega del Carmen, *NVS* Usaime, *SS* Santa Rosa

^a^Number of seropositive children

^b^*p* < 0.0033 is significant at 95% CI (0.05 significance level/15 pairwise comparisons)

Children’s travel was assessed to determine their potential exposure to regions with *B. bacilliformis* infection. From the data in Table 1, respondent children were combined into two groups and compared for seropositivity: (i) those who reported no travel or travel to non-endemic regions of Ecuador (88.1%) and (ii) children who traveled to endemic regions in Ecuador or Peru (8.4%). Of the children who traveled to endemic areas in Ecuador or Peru, 38.9% (7/18) were seropositive compared to 12.5% (3/24) of children who reported no travel or travel to non-endemic areas in Ecuador (data not shown). A majority of respondent children (57%) identified a picture of a sand fly and 86% said they were bitten by sand flies (Table 1). When asked if they could distinguish sand flies from mosquitos, most children and adults answered yes.

Household variables are summarized in Table 3. The majority of surveyed houses were open constructions with adobe walls (86%), dirt floors (72%), and tile roofs (92%). Dogs (90%) and cats (63%) were present at most residences, but usually lived outside the residence and were free-roaming. The most abundant livestock were chickens (95%) followed by pigs (44%), guinea pigs (44%), and goats (27%). Guinea pigs were a food source and caged in the cooking area within the house while chickens were kept in nesting boxes attached to an outside wall of the house. Pigs and goats were kept in small corrals or staked near the house. Stored grains and foodstuffs were stored inside houses and attracted rats and mice to 88% and 84% of residences, respectively. Opossums and foxes were common near 69% of residences. Construction materials such as lumber (14%) and rocks or bricks (34%) were piled within 5–15 meters of houses. Seventy-six percent of households had woodpiles for cooking. Piles of organic debris including compost, crop waste, and animal manure were commonly located 5–10 meters from residences. Wild-growing vegetation (non-identified weeds and bushes) were abundant around most houses, while 51% had cultivated mango or banana trees (Table 4).

**Table 4:** Household characteristics in the six study communities in Loja province

Households	*n*	%
Type of roof		
Tile	106	92.2
Concrete	4	3.5
Zinc	3	2.6
Asbestos	1	0.9
Palm Straw	1	0.9
Type of walls		
Adobe	110	86.1
Brick/concrete	17	13.0
Bamboo/paper lined	1	0.9
Type of floor		
Dirt	83	72.2
Concrete/brick	25	21.7
Rough wood	6	5.2
Bamboo	1	0.9
Peridomestic animals		
Dogs	102	89.5
Cats	72	63.2
Birds/Livestock		
Chickens	110	95.6
Pigs/swine	95	83.3
Guinea pigs	51	44.3
Sheep/goats	31	27.1
Wild animals in/near house		
Mice	97	84.3
Rats	100	88.7
Opossums/foxes	69	60.5
Materials near house		
Firewood	87	76.3
Trash	79	68.7
Rocks/bricks	39	34.2
Lumber pile	16	14.0
Organic matter		
Animal manure	42	36.8
Crop waste	37	32.2
Vegetation		
Bushes	93	81.2
Weeds	83	72.8
Fruit trees	51	44.3
Agave	1	0.9

Large numbers of demographic, household, and environmental variables were evaluated for their risk association with *B. bacilliformis* infection in this study. Many of the variables in the questionnaires were relevant to sand fly habitat and attractants that would affect children’ exposure to sand flies and *B. bacilliformis* (Tables 1 and 4). These variables were individually assessed for their association with seropositivity using univariate analysis. A summary of the univariate analysis is shown in Table 5. There was increased risk for seropositivity with the presence of sheep/goats (OR = 2.06, *p* *=* 0.027) and lumber pile (OR = 2.00, *p* *=* 0.052) and lower risk of seropositivity with presence of weeds (OR = 0.43; *p* *=* 0.012) and incremental 100 m increases in elevation (OR = 0.79, *p* *<* 0.001).

**Table 5:** Univariate associations between *B. bacilliformis* seropositivity and select variables

Variable	Seropositive *n* (%)	Seronegative *n* (%)	OR (95% CI)	*p*
Recognize sand flies				
Yes	56 (64.4)	113 (54.3)	1.52 (0.88–2.63)	0.122
No	31 (35.6)	95 (45.7)		
Type of walls				
Adobe	73 (91.2)	171 (82.2)	2.26 (0.74–6.87)	0.113
Brick/concrete	7 (8.8)	37 (17.8)		
Guinea pigs				
Yes	40 (49.4)	78 (37.5)	1.63 (0.87–3.02)	0.115
No	41 (50.6)	130 (62.5)		
Sheep/goats				
Yes	28 (34.2)	42 (20.1)	2.06 (1.06–4.01)	0.027^a^
No	54 (65.8)	167 (79.9)		
Rats				
Yes	67 (81.7)	185 (89.4)	0.53 (0.24–1.19)	0.122
No	15 (18.3)	22 (10.6)		
Animal waste				
Yes	23 (28.0)	85 (40.7)	0.57 (0.30–1.09)	0.071
No	59 (72.0)	124 (59.3)		
Lumber pile				
Yes	15 (18.3)	21 (10.0)	2.00 (0.99–4.05)	0.052^a^
No	67 (81.7)	188 (90.0)		
Weeds				
Yes	48 (59.3)	161 (77.0)	0.43 (0.22–0.55)	0.012^a^
No	33 (40.7)	48 (23.0)		
Elevation (m)^b,c^	–	–	0.79 (0.70–0.89)	<0.001^a^
Mean	1326 (*n*=89)	1448 (*n*=228)	–	<0.001^a^

*OR* odds ratio, *CI* confidence interval

^a^Statistically significant at *p* < 0.05. Odds ratio > 1.0 denotes increased risk

^b^As a variable, elevation is continuous and thus has no entries for number or % of seropositive/seronegative. However, a univariate odds ratio using logistic regression could still be calculated. The odds ratio is 0.79 for each 100 m of elevation and *decreases* for each 100 m *increase* in elevation

^c^Because elevation is continuous, we also calculated and compared the mean elevation for seropositives and seronegatives by Student’s *t*-test

For the multivariate analyses, variables in the univariate model with values *p* < 0.10 (Table 5) were investigated for more complex associations with *B. bacilliformis* seropositivity that might not have been evident with the univariate analysis. These variables included animal waste (*p* *=* 0.071), sheep/goats (*p* *=* 0.027), lumber pile (*p* *=* 0.052), weeds *(p* *=* 0.012), and elevation (*p* *<* 0.001). Two different multivariate models using logistic regression were tested. The first model examined relationships between seropositivity and the five variables listed above (CI = 95%) and found increased risk with lumber pile and decreased risk with increasing elevation (data not shown). Having sheep/goats was a risk factor for seropositivity in the univariate model, but dropped out in the first multivariate model. The second multivariate model was a refinement of the first model and tested three variables (animal waste, lumber pile, and elevation) (Table 6). There was decreased risk for seropositivity with each 100 meter increase in elevation (OR = 0.76; *p* *<* 0.001) and the presence of animal waste (OR = 0.47; *p* *<* 0.026). Increased risk for seropositivity was observed for the presence of a lumber pile*s* (OR = 2.47; *p* *<* 0.026). To summarize the overall results of both the univariate and multivariate models, having a lumber pile increased the risk of being antibody seropositive and decreased risk was observed with each 100 m increase in elevation and with the presence of animal waste.

**Table 6:** Multivariate model 2 using logistic regression to determine associations between *B. bacilliformis* seropositivity including statistically significant variables from univariate analysis

Variable^a^	Odds Ratio (95% CI)	*p* ^b^
Animal waste	0.47 (0.24, 0.91)	< 0.026
Lumber pile	2.47 (1.08, 5.63)	< 0.032
Elevation (per 100 m)^c^	0.76 (0.66, 0.87)	< 0.001

*OR* odds ratio, *CI*  confidence interval

^a^Animal waste was also included for biological importance as a sand fly breeding site and larval food source

^b^Statistically significant at *p* < 0.05

^c^The odds ratio is 0.76 for each 100 m of elevation; the odds ratio *decreases* for each 100 m *increase* in elevation. This is consistent with the univariate analysis

## Discussion

This is the first study reporting the seroprevalence of *B. bacilliformis* infection in children in the mountain highlands of Loja province, Ecuador. The study area borders provinces in Ecuador and Peru where sporadic outbreaks of Oroya fever were reported over 20 years ago, but is more distant to the lowland coastal provinces of Guayas and Manabi in western Ecuador where verruga peruana is more prevalent.

While the scope of this study did not include investigating clinical cases of Carrion’s disease (Oroya fever and verruga peruana), our results indicate a high seroprevalence for *B. bacilliformis* infection in the children tested. This *Bartonella* study ran in parallel with an ongoing study of Chagas disease in Ecuador. A physician with the Chagas study inquired about Carrion’s disease in the six study communities as well as in Cariamanga and Catachocha in Loja province. Local medical personnel were vaguely, or not aware of Carrion’s disease or illness caused by *B. bacilliformis* and did not report treating anyone with bleeding skin lesions or febrile illness suggestive of Oroya fever (high fever with severe anemia and fatigue). At the time of this study, we were not aware of any public health surveillance of Carrion’s disease in Loja province by the Ecuadorian Ministry of Health. However, we located a physician in Portoviejo, Manabi who reported previously treating patients with verruga peruana

In the study-sponsored clinics, parents were questioned and did not recall any prior serious illnesses in their children with clinical signs indicative of Oroya fever or verruga peruana such as high fever, joint and muscle pains, extreme fatigue, or “bleeding warts”. Likewise, in “endemic” regions of Ecuador that included the lowland provinces of Manabi and Guayas and the highlands of Zamora-Chinchipe (1987–1995), seroprevalence ranged from 11%–21%, respectively, in asymptomatic contacts of patients who were sick with verruga peruana or Oroya fever^28^.

This suggested that infections with *B. bacilliformis* were probably mild and asymptomatic or symptoms confused with other illnesses. Similar findings have been reported in Peru where 56% of seropositive individuals were asymptomatic in a non-endemic area^5^ and 9–29% of infections were asymptomatic in an endemic area^6,13^. In a contemporary study in Piura, Peru, people previously diagnosed with acute Carrion’s disease were followed up a year later and although healthy appearing, many reported mild symptoms with 38.9% positive for *B. bacilliformis* by real time PCR and 25.4% seropositive with IFA titers > 256^30^.

Thirty years earlier, Amano et al.^28^ reported “monophasic verruga peruana” in several communities in coastal Manabi, Ecuador (Portoviejo, Pajan, Jipijapa, and Sucre) with analysis of 16S rRNA gene fragments from skin lesions showing 96% percent identity to the 16S rRNA fragment of type strain *B. bacilliformis* KC583, thus suggesting the emergence of a less virulent strain of *B. bacilliformis* in Ecuador. We characterized a patient isolate of *B*. *bacilliformis*, EC-01 (NCBI accession numbers *gltA* DQ179109 and ITS DQ179107), that was associated with acute splenomegaly and anemia in an expatriate Ecuadorian who visited a non-endemic area of northern Ecuador three years prior to the onset of symptoms^29^. EC-01 had 99–100% sequence identify to *gltA* gene fragments from a majority of *B. bacilliformis* patient isolates from endemic Caraz, Ancash, Peru, but only 96–98% sequence identity with four other isolates from the same area. IFA titers were 256–1028, respectively, when screened against antigens from two other patient isolates from Caraz, Ancash, Peru^29^. Most recently, a novel *Bartonella* agent was isolated from blood of three children with verruga peruana in Caraz, Ancash Peru and determined by MLST analysis to be a new species, Candidatus *B. ancashensis*^31,32^.

Seropositive children in the six study communities had an rPap31 ELISA-IgG GMT of 152, a relatively high titer in the absence of clinical disease. The higher antibody titers in our study may be due to higher seroreactivity with purified rPap31 antigen than with the whole-cell antigens used in IFA. There may also be higher circulating antibody titers due to bacteremia with chronic, subclinical *B. bacilliformis* infections, as has been observed with *B henselae*-mediated bacteremia in cats^33,34^ and bacteremia in blood samples from asymptomatic blood donors in Brazil that tested positive for *B. henselae* (15/500) and *B. clarridgeiae* (1/500) by serology and enrichment PCR^35^. Seroprevalence to *B. bacilliformis* infection in the absence of diagnosed Carrion’s disease further suggests that less virulent or attenuated strains of *B. bacilliformis* may be circulating in the region^36^. It is also possible that transmission of *B. bacilliformis* by alternative arthropod vectors like ticks^37^ or other species of *Lutzomyia* sand flies with lower elevational habitats in the Pacific coastal lowlands or Amazonas regions of Ecuador might attenuate virulence of the pathogen and result in milder infection^14^. Recently, a novel species, Candidatus *Bartonella rondoniensis* was described in *Eratyrus mucronatus* (kissing bugs) in French Guyana and is closely related phylogenetically to the Peruvian isolates *B. ancashensis* and type strain *B. bacilliformis* KC583 by MLST analysis^38^. *E. mucronatus* is a sylvatic species of triatomine that is naturally associated with bats and invasive to human domiciles, and present in some coastal areas in Ecuador as well as in the Peruvian Amazon^39–41^. It is important to investigate the prevalence of *E. mucronatus* in Ecuador and determine whether Candidatus *B. rondoniensis* is present, particularly in regions of Ecuador with verruga peruana and subclinical or asymptomatic infections attributed to *B. bacilliformis*.

Several epidemiological studies of Carrion’s disease in children in Peru have reported that pediatric populations aged 1–14 years of age are the most commonly affected groups, particularly children < 4 years of age^6,42^. Our data support these observations with seropositivity in children aged 1–14 ranging from 12–47% and a peak seropositivity observed in children 3–5 years of age (Fig. 1).

While all six of the communities in this study were located in the altitudinal range for *Lutzomyia verrucarum* (600–3200 m above sea level), both univariate and multivariate regression models showed an inverse relationship between seropositivity and elevation in which risk for seropositivity decreased with each 100 m increase in elevation. Changes in seropositivity within the zonal range for sand flies might be associated with microclimates as elevation decreases such that sand flies are protected from the wind, especially since they are poor fliers^43^. If so, living in a house at a sheltered lower elevation instead of on a windy mountaintop might increase exposure to sand flies infected with *B. bacilliformis*.

Nocturnal *L. verrucarum* (sand flies) prefer relatively undisturbed nesting sites that are cool, humid, and dark such as in soil and rock crevices, between tree buttresses, under loose tree bark, tree holes, caves, animal burrows, and around human dwellings in piles of rocks or wood^44,45^, which might explain the increased risk of lumber piles for seropositivity observed in both the univariate and multivariate models. In contrast to woodpiles that are used for cooking and constantly used and replenished, lumber piles for construction might remain undisturbed for longer periods of time and provide good nesting sites for sand flies.

Sand flies breed near humid, terrestrial habitats where there is a good source of organic matter such as animal manure and vegetative wastes in which to deposit eggs, and on which larvae feed^43,45^. Thus, the presence of animal and vegetative waste would be expected to increase risk. The observed lower risk for seropositivity with animal waste might result if chickens and other birds were pecking through the manure and eating sand fly larvae, thus reducing sand fly populations near residences and the risk of infection with *B. bacilliformis*.

We were surprised that neither rodents nor chickens were risk factors for seropositivity in either the univariate or multivariate models and that sheep/goats dropped out as a risk factor in the multivariate model. In Zamora-Chinchipe, dead rodents were found more frequently in houses with confirmed cases of acute Oroya fever^27^, and sick or dead chickens were found in households with human cases of verruga in Manabi^4^. However, the only known vertebrate reservoir for *B. bacilliformis* are humans^46^. Since livestock and chickens are kept near residences and would attract sand flies and provide blood meals, these animals could be potential reservoirs for *B. bacilliformis* infection and should be investigated in future studies.

The results of this study suggest that exposure to *B. bacilliformis* may be more geographically widespread in Ecuador than historical reports indicate and that this pathogen may be a significant unaddressed cause of childhood illness. However, more studies are required to understand the biology of the sand fly vector(s), potential animal reservoirs, associations between antibody seropositivity to *B. bacilliformis* and clinical disease, and genetic relationships between *B. bacilliformis* strains circulating in different regions of Ecuador and to strains circulating in Peru.

## Materials and methods

### Study area

**Fig. 2:**
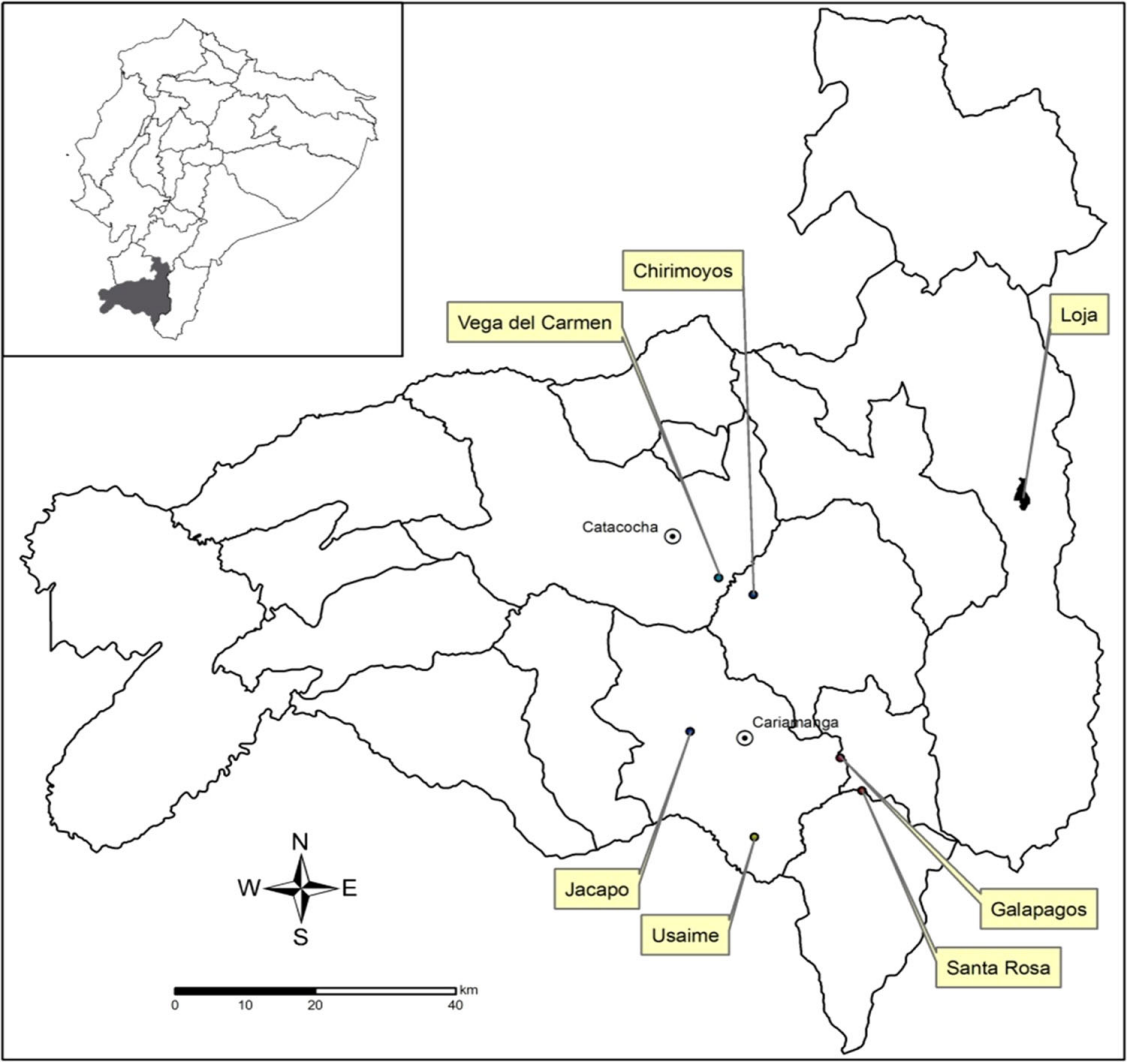
Map of Ecuador (inset) with Loja province shaded. The six study communities are identified in the large map of Loja province. Also shown is the provincial capital city of Loja and major communities of Catacocha (Paltas County) and Cariamanga (Calvas County). WGS84 coordinates for each community are as follows: Chirimoyos (04.12972°S, 79.5456°W), Vega del Carmen (04.10610°S, 79.59012°W), Jacapo (04.36621°S, 79.4162°W), Usaime (04.46035°S, 79.54414°W), Santa Rosa (04.3960°S, 79.41861°W, and Galapagos (04.35233°S, 79.4344°W)

This study was conducted during the dry season between June–August 2009 in Loja Province within the southern Andean Mountains of Ecuador and included the six rural communities. The location of every house in each community was recorded using handheld global positioning system (GPS) units (eTrex, Garmin, Kansas, USA) with World Geodetic System 1984 (WGS84) coordinates. Community locations are as follows: Jacapo (04.36621°S, 79.41620°W) and Usaime (04.46035°S, 79.54410°W) in Calvas County, Galapagos (04.35233°S, 79.43440°W) and Santa Rosa (04.39600°S, 79.41861°W) in Quilanga County, Vega del Carmen (04.10610°S, 79.59012°W) in Paltas County, and Chiromoyos (04.12972°S, 79.54560°W) in Gonzanamá County (Fig. 2). Mean elevations of houses in the communities ranged from 1667 meters in Jacapo to 1112 meters in Chirimoyos (Table 1). Communities were 20–80 km from Zamora-Chinchipe Province in Ecuador and Piura Province in Peru, where Oroya fever was previously reported, and 241–356 km, respectively, from the closest cases of verruga peruana reported in the provinces of Guayas and Manabi in Ecuador. The climate in this region is extremely variable with seasonal temperatures fluctuating between 0–22 °C. Most residences were located on mountain sides or ridge tops, with subsistence farming being the main occupation. Poor living conditions exposed people to a variety of arthropods such as ticks, fleas, mites, mosquitoes, sand flies, and kissing bugs and they resided in close proximity to chickens, livestock, semi-domesticated cats and dogs, rodents, and other wild animals.

### Population and ethics statement

A total of 319 children between the ages of 1 and 15 were enrolled in this study, representing 128 households in six communities (Table 1). Samples were collected at a study-sponsored medical clinic set up in the community school and informed consent was obtained from the parents of minors taking part in the study, in accordance with Ohio University Institutional Review Board (Protocol 07 × 09). Parents and children > 10 years of age responded to an epidemiologic questionnaire related to housing conditions and risk factors. Questionnaires were administered in Spanish by trained field technicians at the clinic^47,48^.

### Blood sampling

Blood was collected by finger stick onto filter paper (Whatman #1, Fisher Scientific) using disposable pediatric Genie lancets (Becton-Dickinson, Franklin Lakes, NJ). Filter paper with samples were placed in individual ziplock bags with desiccant packs and kept at ambient temperature until the team returned each evening to a facility equipped with refrigeration, where samples were stored at 4 °C until use. For long term storage, samples were maintained at –20 °C at the Pontifical Catholic University of Ecuador in Quito.

### Serological analysis

For the elution of antibodies from filter paper, a 5 mm diameter punch was obtained from every dried blood sample and incubated for 2 h at room temperature in 200 µl of 0.5% Tween 20 in phosphate-buffered saline, pH 7.5 (PBST). We used a recombinant protein Pap31 (rPap31) ELISA to measure antibodies specific to *B. bacilliformis*^49,50^. To rule out cross-reactivity of the rPap31 antigen with antibodies to *B. henselae* and *B. quintana*, additional human control sera were tested. Patient sera from IFA-confirmed infections with *B. henselae* and *B. quintana* did not cross-react with rPap31 antigen (titers < 64, data not shown). We also tested archived sera (*n* = 15) from healthy male employees of the Servicio Nacional de Control de Enfermedades Transmitidas por Vectores Artrópodos (SNEM) from Loja and Manabi provinces who had occupational exposure to cats, dogs, wild animals, fleas, ticks, and sand flies that might be infected with *Bartonella*. With both the rPap31 ELISA and three *Bartonella* IFAs^51^, 15/15 sera were antibody-negative for *B. quintana* and *B. henselae* (titers < 64) while 10/15 were antibody-positive for *B. bacilliformis* (data not shown). Although we could not unequivocally rule out the presence of antibodies to other *Bartonella* species in childrens’ sera, based on the results of these serological assays, we were confident that the rPap31 ELISA was measuring antibodies specific to *B. bacilliformis* and not cross-reacting to those from antigenically related bartonellae.

Ninety-six well microplates (Costar 3597, Corning, Lowell, MA) were coated overnight at 4 °C with 0.3 µg/well rPap31 antigen prepared in carbonate buffer (0.5 M Na_2_CO_3_/NaHCO_3_, pH 9.5) and blocked with 5% nonfat milk in PBS (Blotto, Thermo Scientific, Massachusetts,USA) for 60 min at room temperature. Eluates were diluted 1:32 in Blotto, then added to the antigen-coated plate, serially diluted two-fold, and incubated at 37 °C for 60 min. All samples and controls were assayed in duplicate. Plates were washed 3 × with PBST and 1 × with PBS. Goat anti-human IgG conjugated to biotin in 5% BSA in PBS (1:4000) was added to plates and incubated for 60 min at 37 °C. After washing, plates were incubated with horseradish peroxidase-conjugated to streptavidin (1:4000) with 5% BSA in PBS for 60 min at 37 °C. Following washing, plates were incubated with 1-step Ultra TMB-ELISA substrate (Pierce, Rockford, IL) for 15 min at room temperature, the reaction stopped with 1% SDS, and plates read at 405 nm using a Spectramax 190 spectrophotometer and Softmax Pro software (Molecular Devices; Sunnyvale, CA). Each plate contained positive, negative, and blocking controls. The positive control was antisera from an Ecuadorian patient with acute Oroya fever (titer 1024). Negative controls were sera obtained from regional blood banks throughout Ecuador and confirmed by IFA to be antibody-negative for *B. henselae*, B. *quintana*, and *B. bacilliformis* (titers < 64). The optical density (OD) of positive and negative controls were used to determine the limits for seropositivity and seronegativity of the assay using a statistical method^52^. Briefly, an upper prediction limit for negative control replicates was calculated using a Student-*t*-distribution and the standard deviation was multiplied by a factor based on the number of negative controls and the confidence level (1-α), which was 95% CI in this study. IgG titers > 64 were scored positive for antibodies to *B. bacilliformis*. The mean OD_405_ for negative control sera was 0.0571 and the OD for antibody-positive samples was greater than 0.541 in the rPap31 IgG ELISA.

### Data management and analysis

Statistical analyses for the demographic data were performed using SAS software package version v9.2 (Cary, NC) and R analysis. All statistical analyses accounted for anticipated within-household clustering by using survey sampling analytic methods for cluster sampled data. Descriptive statistics were computed for all variables of interest for comparison purposes and to examine distributions for fit to statistical assumptions. The associations between seropositivity and age, gender, and community were evaluated using tests for independence such as Pearson’s Chi-square, Cochran-Mantel-Haenszel tests, and Fisher’s exact test.

Univariate analyses were performed between seropositivity and other demographic and epidemiologic variables using the Rao-Scott likelihood ratio chi-square test for nominal level variable comparisons (yes/no), and comparison of means by *t*-test for continuous variables (elevation). Odds ratios (ORs) and 95% confidence intervals were also estimated for bivariate (two-level) variables and elevation (continuous), and considered statistically significant for *p*-values < 0.05. Variables that were first tested in univariate analysis and had *p*-values < 0.10 (animal waste, sheep/goats, lumber pile, weeds, and mean elevation) were analyzed using multivariate logistic regression models to test their independent relationships with *B. bacilliformis* seropositivity.
